# Influence of Marriage upon Health

**Published:** 1875-01

**Authors:** 


THE INFLUENCE OF MARRIAGE UPON HEALTH. M. Bertillon lately having had to draw up a paper for the Academy of Medicine of Paris, on the influence of marriage on mortality, consulted the registers of only three countries in Europe which were carefully enough kept to give him a reply to his question, those of France, Belgium, and Holland, and shows that if the male sex be first considered, we find that from 25 to 30, 1,000 married men furnish 6 deaths; 1,000 unmarried, 10 deaths; and 1,000 widowers, 22 deaths. From 30 to 35, of 1,000 married men, 7 die; of 1,000 unmarried men, 11½ die; and of 1,000 widowers, 19 die. From 35 to 40, of 1,000 married men, 7½ die; of 1,000 bachelors, 13 die; and of 1,000 widowers, 47½ die; and so on at all the following ages, the married man continues to live with greater facility than the bachelor. It has been that since the most fortunate men can afford to marry, it is not astonishing that these persons should live longer. But this will not, of course, account for the very great mortality of widowers at all ages, which, indeed, surpasses that even of bachelors.

However, it must be noticed that 8,000 young men marry in France yearly, under the age of 20. This is very fatal to such young men, for M. Bertillon finds that, whilst 1,000 young men from 15 to 20 furnish 7 deaths, when unmarried, no less than fifty deaths occur among 1,000 young married men under 20, Hence *premature* marriage is the surest way to old age.

Women seem to reap less advantage from marriage than men, and there is but little difference in the mortality of unmarried and married women before the age of 25. It is but little marked even between 25 and 30. From 30 to 35, out of 1,000 spinsters, 11 die; and of 1,000 wives, 9 die. This difference augments up till 55 years of age. Thus, from 50 to 55 years of age, 1,000 wives have 15 to 16 deaths a year, whilst 1,000 spinsters or widows have 26 to 27 deaths. This advantage rests very marked beyond this age, although it diminishes a little ; but before 25 years of age, and before 20 in Paris, marriage, far from being favorable to the life of women, is injurious to it. The mortality of maidens from 15 to 20 is 7.53 per 1,000; of wives of the same age, is 11.86 per 1,000; and the mortality of married women from 20 to 25 being 9.92 per 1,000, the mortality of maids of the same age is 8.32.

With respect to widowhood, from 25 to 30, widowhood is injurious to health; and whilst 1,000 wives and maids of this age furnish 9 deaths a year, 1,000 widows have 17 deaths.

But in France, and especially at Paris, this mortality quickly diminishes, and at 45, the death-rate of widows is lower than that of spinsters at the same age. And at this age women who have had children are least attacked by death. "Thus marriage in women retards old age and soothes its sufferings."

The conclusion of these statistics is that marriage is an element of health much more powerful than has been supposed, that it exercises its salutary influences especially on the male in the age of vigor, and on the female in her declining years. A calculus of probabilities shows that the man who marries between 20 and 25 has 40 years to live in place of 35, and that the young woman who marries between 20 and 25 has 40 in place of 36. Criminality, suicide, and insanity are far less common among the married than the single.

---

THE ENVELOPE.—Enclose your subscription for next year. Add *five cents* for postage.

